# A Preliminary Investigation of Embedding In Vitro HepaRG Spheroids into Recombinant Human Collagen Type I for the Promotion of Liver Differentiation

**DOI:** 10.3390/polym14091923

**Published:** 2022-05-09

**Authors:** Fang-Chun Liao, Yang-Kao Wang, Ming-Yang Cheng, Ting-Yuan Tu

**Affiliations:** 1 Department of Biomedical Engineering, National Cheng Kung University, Tainan 70101, Taiwan; qqmellow@gmail.com (F.-C.L.); ohyougotmee@gmail.com (M.-Y.C.); 2 Department of Cell Biology and Anatomy, College of Medicine, National Cheng Kung University, Tainan 70101, Taiwan; humwang@mail.ncku.edu.tw; 3 Medical Device Innovation Center, National Cheng Kung University, Tainan 70101, Taiwan; 4 International Center for Wound Repair and Regeneration, National Cheng Kung University, Tainan 70101, Taiwan

**Keywords:** HepaRG, 3D culture, spheroid, recombinant human collagen, in vitro liver model

## Abstract

Background: In vitro three-dimensional (3D) hepatic spheroid culture has shown great promise in toxicity testing because it better mimics the cell–cell and cell–matrix interactions found in in vivo conditions than that of the traditional two-dimensional (2D) culture. Despite embedding HepaRG spheroids with collagen type I (collagen I) extracellular matrix (ECM) revealed a much better differentiation capability, almost all the collagen utilized in in vitro hepatocytes cultures is animal-derived collagen that may limit its use in human toxicity testing. Method: Here, a preliminary investigation of HepaRG cells cultured in different dimensionalities and with the addition of ECM was performed. Comparisons of conventional 2D culture with 3D spheroid culture were performed based on their functional or structural differences over 7 days. Rat tail collagen (rtCollagen) I and recombinant human collagen (rhCollagen) I were investigated for their ability in promoting HepaRG spheroid differentiation. Results: An immunofluorescence analysis of the hepatocyte-specific functional protein albumin suggested that HepaRG spheroids demonstrated better hepatic function than spheroids from 2D culture, and the function of HepaRG spheroids improved in a time-dependent manner. The fluorescence intensities per unit area of spheroids formed by 1000 cells on days 7 and 10 were 25.41 and 45.38, respectively, whereas almost undetectable fluorescence was obtained with 2D cells. In addition, the embedding of HepaRG spheroids into rtCollagen and rhCollagen I showed that HepaRG differentiation can be accelerated relative to the differentiation of spheroids grown in suspension, demonstrating the great promise of HepaRG spheroids. Conclusions: The culture conditions established in this study provide a potentially novel alternative for promoting the differentiation of HepaRG spheroids into mature hepatocytes through a collagen-embedded in vitro liver spheroid model. This culture method is envisioned to provide insights for future drug toxicology.

## 1. Introduction

The liver is an essential organ for the detoxification of various metabolites. Hepatotoxicity caused by drugs usually causes serious human health problems, such as acute liver failure. Developing in vitro liver models with tissue-like functionality may provide insights to advance disease modeling and drug discovery [1]. Cell spheroid culture has recently received significant attention for the development of in vitro models. Compared with conventional two-dimensional (2D) cell culture, three-dimensional (3D) spheroids provide an improved cell culture environment that fosters cell–cell and cell–matrix interactions similar to those found in the in vivo conditions [2,3,4,5,6,7]. Engineered liver tissues of the digestive system could be mimicked using hepatic spheroids generated from primary human hepatocytes (PHHs) that showed metabolic and synthetic functions similar to those of functional hepatocytes in the liver, highlighting the great potential of this system in tissue engineering for liver regeneration [8].

Among the different types of cells available, PHHs are the gold standard for establishing an in vitro liver model. However, under the in vitro maintenance of PHHs in 2D culture, the cells rapidly lose their phenotypic functions [9]. In addition, this model is considered a less economical model than other models, with large sample-to-sample variabilities due to the different sources of donors [10]. Hence, human-derived hepatic cell lines that retain their phenotypic functions could be a valuable alternative to PHHs for drug metabolism and toxicity studies. In contrast to 2D-cultured PHHs, spheroids generated from the HepaRG cell line allow accurate assessment of acute toxicity similar to in vivo hepatotoxicity after maintenance for 3 weeks [11]. In addition, HepaRG cells can express physiologically relevant levels of various cytochrome P450 enzymes, which are important in human drug metabolism [12,13,14].

Various approaches for the formation of hepatic spheroids have been proposed and are generally categorized into scaffold-free or scaffold-based methods [8,15]. While scaffold-free culture shows comparative ease of implementation through enhanced cell–cell interaction rather than cell–substrate interaction, scaffold-based approaches integrating hydrogels/extracellular matrix (ECM) have become increasingly popular due to their high water content and tissue-like physiochemical properties [16]. For instance, Fong et al. developed a cellulosic microporous hydrogel sponge that enables functional hepatic cell culture and retrieval, rapid differentiation enhancement, and drug testing [17]. Their results showed that the differentiation of HepaRG spheroids cultured with a proper scaffold or ECM could be accelerated to 12 days, in contrast to the 28 days achieved in 2D culture without an ECM. However, the gel material in the study was prepared explicitly by the research group and is not available for general use. In addition, the material is different from native ECM, and the preparation procedure is sophisticated. Given that there is currently no consensus concerning the multiple variables of spheroid culture condition, including the diameter, seeding cell density, incubation time, and addition of ECM, there hence remains ample room for improvements, and further investigations of spheroids and their culture conditions with ECM are warranted.

Collagen type I (collagen I), the most frequently found protein in liver ECM [18], plays a major role in cell growth, differentiation, and survival and tissue organization [19]. In vitro assays have commonly applied collagen I coating/substrate in 2D cultures or have entrapped hepatocytes in a sandwich configuration between two layers of collagen I, as an alternative 3D model [20,21]. To the best of our knowledge, only two recent studies attempted embedding hepatic spheroids (PHH or HepaRG) into collagen I to achieve a more advanced and optimized 3D culture model [22,23]. In addition, almost all of the collagen utilized in the in vitro hepatocytes cultures is animal-derived collagen, mainly bovine, porcine, and rat tail [21,24,25], which may increase the risks of transmissible viruses and diseases [26]. Besides, purification and variation between batches may lead to poor predictions for in vitro toxicity testing [27]. In contrast, recombinant human collagen (rhCollagen) I, acquired by expressing a specific gene segment transcribed into the host with specific-pathogen free condition, may serve as a reliable source for reproducible and predicable ECM for hepatocyte culture.

In this study, in vitro hepatic spheroid cultures using both scaffold-free and scaffold-based methods were investigated for their ability to promote liver differentiation. The conventional 2D culture was compared with 3D spheroid culture with respect to their functional or structural differences using HepaRG normal liver cells. Morphological differences among traditional 2D culture, 3D spheroid culture, and 3D spheroid culture with the addition of collagen I were observed. ECM provided dimensionality, and spheroids grown in suspension or embedded into rat tail collagen (rtCollagen) I or rhCollagen I were investigated. The assay was validated via immunofluorescence, quantitative polymerase chain reaction (qPCR) and the expression of human serum albumin was measured. In short, the proposed platform can be used to provide insights for the development of an in vitro liver model and drug toxicology.

## 2. Materials and Methods

### 2.1. Cell Maintenance

HepaRG cells are cells of an immortalized cell line that possess the specific characteristics of primary human hepatocytes. Cells of this cell line were kindly provided by Prof. H. Sunny Sun at the Institute of Molecular Medicine at National Cheng Kung University, Tainan, Taiwan, and maintained in Williams’ Medium E Basal Medium consisting of GlutaMAX supplemented with serum-free induction medium supplement (HPRG750; Gibco, Gaithersburg, MD, USA). The cells were cultured in T25 flasks in medium supplemented with 10% fetal bovine serum (Gibco) and 1% penicillin-streptomycin, and grown in the presence of 5% CO_2_ at 37 °C in a humidified incubator. The medium was replaced with fresh medium three times per week. Before HepaRG cell seeding, the T25 flask was immersed in 0.02 N acetic acid with a collagen concentration of 100 μg/mL for 30 min to enhance cell attachment to the flask and then washed twice with phosphate-buffered saline (PBS; Invitrogen, Carlsbad, CA, USA).

### 2.2. Spheroid Culture

Spheroids were generated by the gravity-forced aggregation of cells in ULA 96-well plates (7007; Corning, Corning, NY, USA). Cells at the desired densities of 1000 cells per well and 5000 cells per well were seeded in a 96-well plate. The culture media were refreshed every 2 days by removing 100 μL of the medium (from a total medium of 200 μL per well) and adding 100 μL of fresh medium, and the spheroids were maintained for 8 days in culture. The HepaRG spheroids obtained with different cell concentrations and over time during their formation were observed with an inverted microscope (CKX53; Olympus, Tokyo, Japan). Image analysis was performed using ImageJ (NIH, Bethesda, MD, USA) to calculate the volume and circularity of the spheroids. The radii (r) of the spheroids were obtained from ImageJ software analysis, and volume (V) was calculated based on the volume of a sphere using $V=\frac{4}{3}\pi r^{3}$.

### 2.3. Embedding of Spheroids into Collagen

The stiffness of the ECM in fresh human tissue corresponds to the collagen type I concentration, which ranges from 1.4 to 2.3 mg/mL [28]. In this study, a final concentration of collagen type I solution of 2 mg/mL was prepared. This collagen type I solution was prepared by mixing rtCollagen I (110101; GEcoll, Tainan City, Taiwan; original concentration 3 mg/mL) or rhCollagen I (211101; GEcoll; concentration 2.5 mg/mL), filter-sterile 10X PBS, 1 N NaOH, and distilled water. Then, 20 μL PBS, NaOH (1.3 μL for the rtCollagen solution and 1.6 μL for the rhCollagen solution), and distilled water (45.4 μL for the rtCollagen solution and 18.4 μL for the rhCollagen solution) were added to the Eppendorf tubes in sequence, and collagen (133.3 μL of rtCollagen and 160 μL of rhCollagen) was added to obtain two types of collagen I solutions. All steps were performed on ice to ensure that the ingredients were mixed well, and the pH of the final solution was tested with a pH test strip to ensure a final pH of 7.4. Twenty microliters of 1000-cell spheroid suspension was added to the collagen I solutions, and the total volume was 220 μL. Twenty microliters of the collagen mixture was distributed equally in each homemade PDMS chamber, and the chamber was incubated at 37 °C for at least 30 min until a solid gel formed. After collagen polymerization, 200 μL of warm (37 °C) culture medium was added carefully along the sidewall onto the gel to cover the collagen/spheroid drops (Supplementary Figure S1).

### 2.4. Live/Dead Staining

The LIVE/DEAD^®^ Viability/Cytotoxicity Assay Kit (Invitrogen) contains two reagents, which provide green/red fluorescent staining for live and dead cells. The cells were incubated with calcein-AM staining solution (1:2000 dilution), EthD-1 staining solution (1:1000 dilution), and Hoechst 33258 nucleic acid staining solution (1:2000 dilution; Invitrogen), which were diluted with PBS, at room temperature for 1 h before imaging examinations for the analysis of cell viability. Images were captured with a fluorescence microscope (IX3; Olympus, Tokyo, Japan) and camera (ISH500, Tucsen Photonics, Fuzhou, China) for fluorescent staining, and ImageJ was used to analyze the percentage areas of live cells and dead cells obtained with the spheroids (Supplementary Figure S2).

### 2.5. Immunofluorescence

For 2D cell immunofluorescence staining, the cells were seeded on glass coverslips. All of the 3D spheroids were collected prior to immunofluorescence staining. For the 3D spheroids embedded in collagen, the spheroids were incubated with 2 µL collagenase in a 37 °C water bath for 5 min to degrade the collagen I, and the spheroids were harvested and collected in Eppendorf tubes. After washing with PBS, the 2D cells and spheroids were fixed with 4% paraformaldehyde (Sigma–Aldrich, St. Louis, MO, USA) at 4 °C for 30 min and washed three times with PBS. PBS containing 0.1% Triton X-100 (Sigma–Aldrich) was added for 30 min, and blocking solution (SuperBlock Blocking Buffer; Thermo Fisher Scientific, Waltham, MA, USA) was then added for 1 h at room temperature. The cells and spheroids were incubated with anti-albumin antibodies (ab207327, 1:400 dilution; Abcam, Cambridge, UK) at 4 °C overnight, washed, and then incubated with the corresponding Alexa Fluor-conjugated secondary antibodies (1:400 dilution), Phalloidin-iFluor 594 (1:5000 dilution) and DAPI (1:5000 dilution) for 1 h at room temperature in the dark. The slides were mounted with glycerol-gelatin (Sigma–Aldrich) after washing. Immunofluorescence images were then obtained using a fluorescence microscope (IX3; Olympus, Tokyo, Japan); the gain value and exposure time for each experiment were held constant. The fluorescence levels were determined by analyzing the fluorescence intensity per unit area using ImageJ.

### 2.6. qPCR

2D HepaRG cells were harvested on day 4 and 3D HepaRG spheroids were harvested at days 7. A portion of the spheroids were harvested at days 4 and cultured three more days in rhCollagen and harvested on Day 7. 3D HepaRG spheroids with collagen were added collagenase in order to break down excess collagen I. 2D cells or 3D spheroids were ground by BioMasher (Power Masher II, Nippi. Inc., Tokyo, Japan). After grinding, the RNA was extracted through RNAspin Mini Kit. The extracted RNA was tested by a spectrophotometer (NanoDrop Technologies, Montchanin, DE, USA) to check the purity and concentration of the RNA, and then the RNA was converted to cDNA through the SuperScript™ IV First-Strand Synthesis System (Invitrogen), and the reverse transcript was mixed with the annealed RNA and heated on a heater at 50 °C for 10 min and 80 °C for 10 min, after which the cDNA was removed and stored in a −20 °C freezer. cDNA was added to the 8-strip tube with the forward primer and reverse primer of albumin and cytochrome P450 family 3 subfamily A member 4 (CYP3A4) and operated at the StepOnePlus™ Real-Time PCR System (Applied Biosystems, Foster City, CA, USA). Initial denaturation was operated at 94 °C for 2 min. After 40 cycles of denaturation, 15 s of annealing was set at 94 °C and 1 min for extension at 60 °C. Template sequences were designed as follows: ALB forward: CCT TTG GCA CAA TGA AGT GGG TAA CC; ALB reverse: CAG CAG TCA GCC ATT TCA CCA TAG G; CYP3A forward: TAA GGA AAG TAG TGA TGG C; CYP3A reverse: CCA GCA CAG GCT GAC C.

### 2.7. Statistical Analysis

Statistical analysis was performed using GraphPad Prism (San Diego, CA, USA) with data expressed as the mean ± standard deviation of at least 2 to 3 replicates. Comparisons among two groups were conducted using an unpaired *t*-test. Comparisons among multiple groups were conducted using one-way ANOVA followed by Tukey’s multiple-comparison test. A *p*-value < 0.05 was considered significantly different, in which * represents *p* < 0.05, ** represents *p* < 0.01, and *** represents *p* < 0.001.

## 3. Results

### 3.1. Morphology Assessment of 2D and 3D Cultures

**Figure 1:**
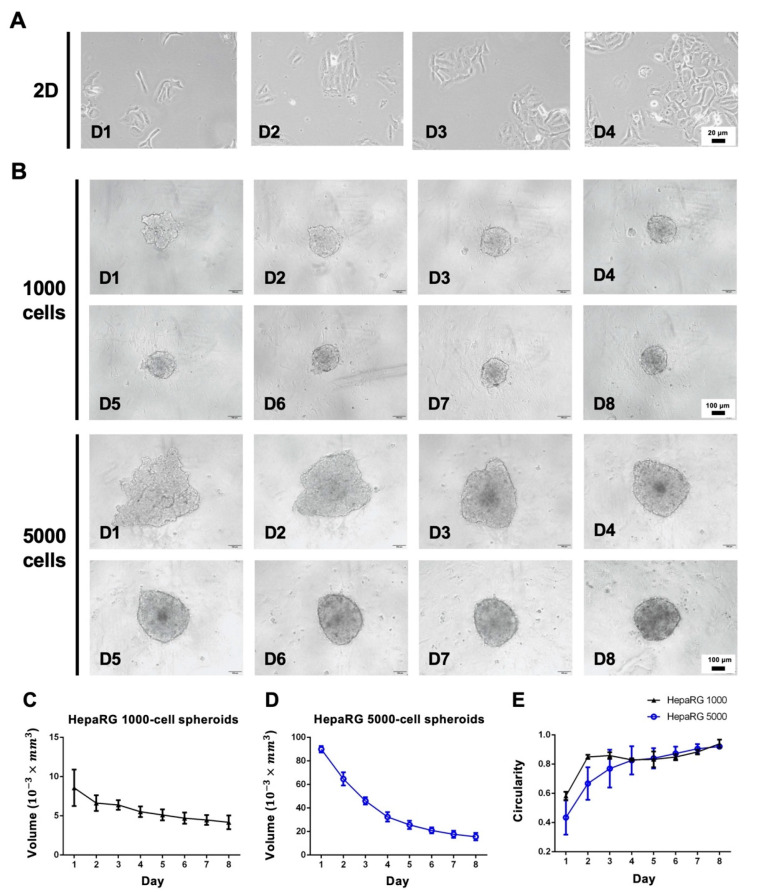
Morphological assessment of 2D and 3D cultures. (**A**) HepaRG cells cultured under 2D conditions. Scale bar, 100 µm. (**B**) Aggregation processes of HepaRG spheroids with different cell concentrations (1000 and 5000 cells per well) over time. Scale bar, 100 µm. (**C**) Quantification of the HepaRG 1000 cell spheroid volume from days 1 to 8. (**D**) Quantification of the HepaRG 5000 cell spheroid volume from days 1 to 8. (**E**) Circularity of spheroids over time.

The first set of experiments involved a conventional 2D culture for the formation of a 3D spheroid (Figure 1). HepaRG cells could be well maintained in 2D culture conditions (Figure 1A). HepaRG spheroids seeded at a density of 1000 and 5000 cells per well in 96-well ULA plates self-aggregated into spheroid-like structures after 3 days of culture (Figure 1B). The shape and size of the spheroids formed by 1000 cells showed no significant changes after 3 days. The diameters obtained were approximately 200 μm, with the volume of the spheroids remaining about 5 (10^−3^ × mm^3^) (Figure 1C). After 6 to 7 days, the diameters obtained were approximately 350 μm in the volume of 5000-cell spheroids tended to be stable at around 20 (10^−3^ × mm^3^) (Figure 1D). Regardless of the initial seeding density, the circularity of the HepaRG spheroids increased over time (Figure 1E).

### 3.2. Viability of 2D and 3D Cultures

**Figure 2:**
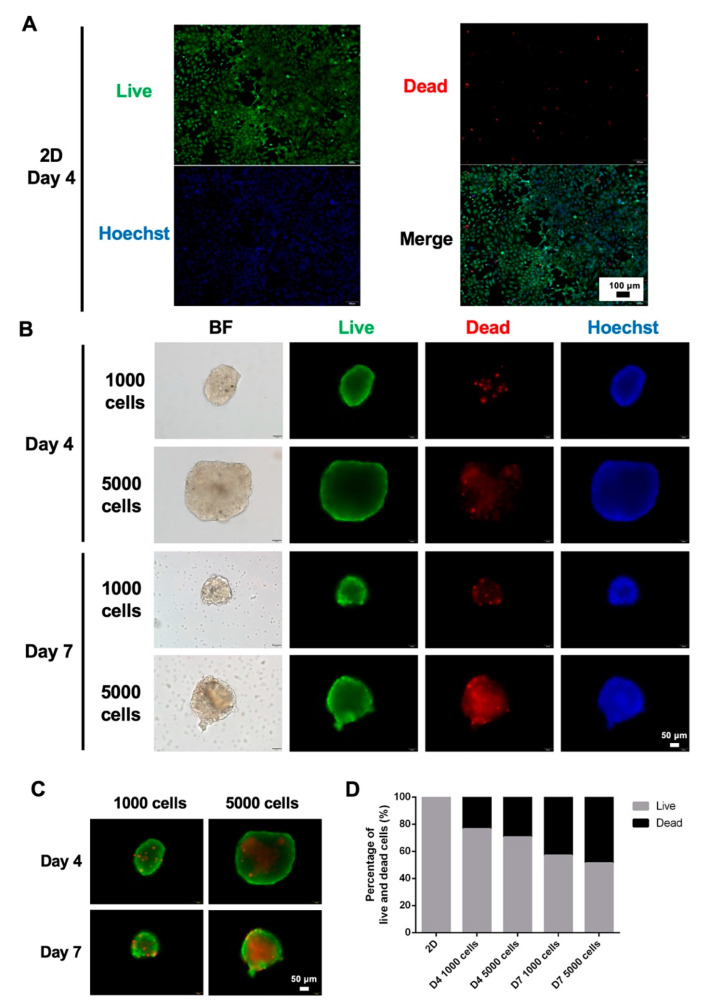
Viability of 2D and 3D cultures. (**A**) Live-cell and dead-cell staining of HepaRG cells under 2D culture conditions for four days. Scale bar, 100 µm. (**B**) Live-cell and dead-cell staining of HepaRG spheroids (seeded at different densities, i.e., 1000 cells and 5000 cells per well) after days 4 and 7 in culture. Scale bar, 50 µm. (**C**) Merged image of live and dead staining of spheroids obtained with different cell seeding densities and after different days of culture. Scale bar, 50 µm. (**D**) Evaluation of the area percentages of live and dead cells of 2D HepaRG cells and 3D HepaRG spheroids.

The viability of 2D cells and 3D spheroids was assessed by live and dead staining (Figure 2). The results showed that most cells exhibited green fluorescence, and only a few scattered dead cells were detected, which indicated that there were very few dead cells after four days of culture under 2D culture conditions (Figure 2A). Under 3D culture conditions, regardless of the culture duration, the dead signal inside the spheroids formed with a seeding density of 5000 cells per well was more pronounced than that obtained with spheroids formed from 1000 cells (Figure 2B), i.e., on day 4, the percentage dead-cell area of HepaRG spheroids formed by 1000 and 5000 cells was 23.66% and 29.55%, respectively (Figure 2C,D). In addition, the spheroids displayed more dead cells in the center on day 7 than on day 4 (Figure 2B). On days 4 and 7, the percentage dead-cell area of HepaRG spheroids formed by 1000 cells was 23.66% and 43.2%, respectively, which suggested a higher dead cell ratio on day 7 than on day 4 (Figure 2C,D).

### 3.3. Structure and Functional Validation of 2D and 3D Cultures

**Figure 3:**
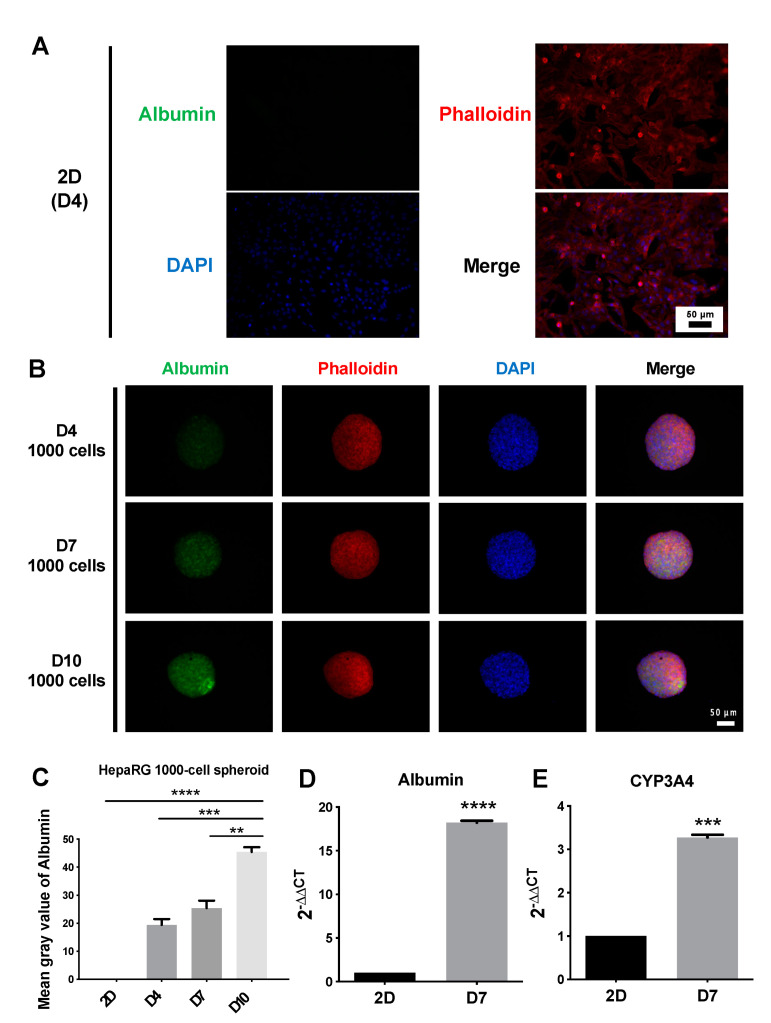
Identification of functional proteins by immunofluorescence staining of albumin (green), phalloidin (red) and nuclei (blue). (**A**) Immunofluorescence staining of HepaRG cells under 2D culture conditions at days 4. Scale bar, 50 µm. (**B**) Immunofluorescence staining of HepaRG 1000-cell spheroids after 4, 7, and 10 days in culture. Scale bar, 50 µm. (**C**) Analysis of the fluorescence intensity of albumin per area obtained with HepaRG 2D cells and 3D spheroids. ** *p* < 0.01, Day 10 vs. Day 7; *** *p* < 0.001, Day10 vs. Day4; **** *p* < 0.0001, Day10 vs. 2D cells. (**D**,**E**) Delta-Delta Ct method for qPCR data analysis of Albumin and CYP3A4 in HepaRG 2D culture and 1000-cell spheroids grown in suspension. The ∆∆Ct is relative to the average of 2D ∆Ct.

To investigate the expression of albumin, a protein that reflects hepatocyte-specific function, in 2D cells and 3D spheroids, immunofluorescence staining was performed (Figure 3). The results showed that under 2D culture conditions, the fluorescence intensity of albumin was very weak and almost undetectable (Figure 3A), and the albumin fluorescence intensity in 3D cultures was higher than that in 2D cultures (Figure 3B). Among the 3D cultures, HepaRG spheroids were seeded at a density of 1000 cells per well. Red fluorescence phalloidin staining can selectively bind to actin filaments. As shown in the merged results (Figure 3B), actin filaments had a dense network structure, and the network structure of actin filaments consisted of nuclei (blue). From the quantitative results, the fluorescence intensity per area of albumin was significantly higher on day 10 (mean gray value of 45.38) than on days 4 and 7 (mean gray values of 19.41 and 25.41, respectively) (Figure 3C). Gene expression results showed that albumin and CYP3A4 expressed significantly higher in 3D spheroids than in 2D culture (Figure 3D,E).

### 3.4. Function of Collagen I in 3D Spheroid Cultures

**Figure 4:**
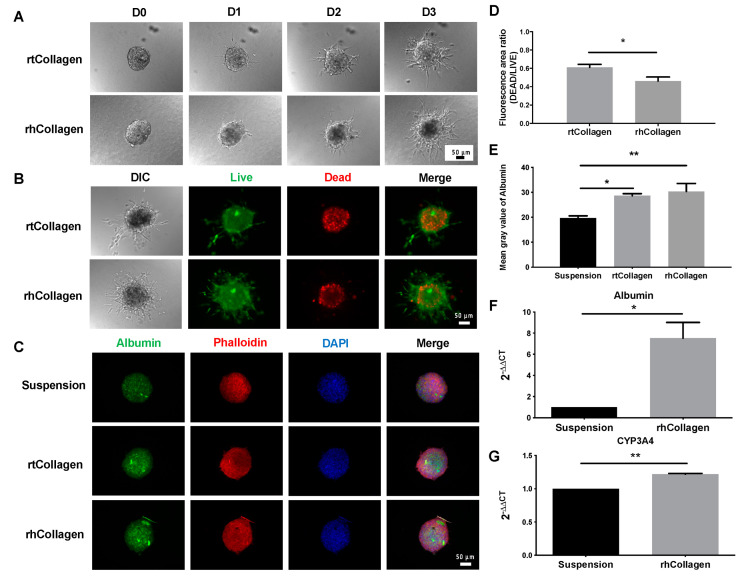
Function of 1000-cell spheroids in suspension or embedded in collagen type I (collagen I) cultures. (**A**) Morphological assessment of HepaRG spheroids embedded in rat tail collagen (rtCollagen) and recombinant human collagen (rhCollagen) and maintained for 3 days. Scale bar, 50 µm. (**B**) Live and dead cell staining of HepaRG spheroids embedded in two types of collagen I for 3 days. (**C**) Immunofluorescence staining of albumin (green), phalloidin (red), and nuclei (blue) of HepaRG spheroids embedded in suspension and two types of collagen I. (**D**) Evaluation of the relative fluorescence of HepaRG spheroids embedded in rtCollagen or rhCollagen showing the area ratio of dead to live cells. (**E**) Quantification of fluorescence intensity per area of albumin in (**C**). * *p* < 0.05, ** *p* < 0.01. (**F**,**G**) Delta-Delta Ct method for qPCR data analysis of albumin and CYP3A4 in HepaRG 1000-cell spheroids grown in suspension and embedded in rhCollagen. The ∆∆Ct is relative to the average of suspension ∆Ct.

Given that collagen I is one of the most frequently found proteins in liver ECM and plays vital roles in liver physiology and pathology [18], the function of collagen I in the promotion of liver function in HepaRG 1000-cell spheroids was investigated (Figure 4 and Supplementary Figure S1). HepaRG 1000-cell spheroids were precultured for 4 days in ULA 96-well plates, and the well-formed spheroids were harvested, mixed with two types of collagen I (rat tail and human recombinant), and maintained for 3 days. During the 3-day experiment, the morphology of the spheroids embedded in collagen was continuously observed. The results showed that HepaRG spheroids could invade two types of collagen I and exhibited a dendritic shape on day 3, highlighting cells leaving the spheroids through these protrusions (Figure 4A). After 3 days of culture, the viability of the embedded spheroids, as determined by live and dead staining, was appropriate (Figure 4B). All embedded spheroids, including the dendritic structure, exhibited green fluorescence (live cells), but bright red signals (dead cells) were observed inside the spheroids. The percentage of the live-cell area of HepaRG spheroids embedded in rtCollagen was 61.81%, and that of spheroids embedded in rhCollagen was 69.88% (Figure 4D). For functional validation, collagen was dissolved to collect the spheroids and then subjected to immunofluorescence staining. The control group consisted of spheroids grown in suspension for 7 days using ULA 96-well plates (Figure 4C). In the merged photographs, nuclei (blue) were tightly surrounded by the network structure of actin filaments (red) (Figure 4C). Based on the quantitative results, the fluorescence intensity per area of spheroids embedded into the two types of collagen I (the mean gray values of rtCollagen and rhCollagen were 28.64 and 30.32, respectively) was higher than that of the suspended control group (mean gray value of 19.74) (Figure 4E). Given that there was no significant difference between the average fluorescence intensity of spheroids embedded into rtCollagen and rhCollagen, gene expression was compared between spheroids cultured in suspension and embedded in rhCollagen. The results showed that albumin and CYP3A4 expressed significantly higher in ECM-supported spheroids than in suspension. (Figure 4F,G)

## 4. Discussion

3D hepatic cellular spheroids provide a unique window for researchers to observe an in vitro model that has part of the physiologically relevant microenvironment because of their improved cell–cell interactions and signaling cascades compared to traditional 2D cell culture. Therefore, the application of spheroid culture has become a crucial topic in the field of tissue engineering and regenerative medicine. An in vitro hepatic spheroid model using HepaRG cell lines not only retains certain morphological features and functions of hepatocytes compared to other liver cell lines, but is also reproducible, and relatively low cost and high throughput compared to the PHH model. Here, we selected ULA 96-well plates and recombinant collagen type I as the spheroid culture and maturation platform to ensure the widest user accessibility and achieve a highly reproducible workflow.

Assessment of morphology and viability during spheroid formation showed that the 3D HepaRG spheroids shrank and formed compact spherical structures over culture time to day 8 (Figure 1B), and the circularity reached 0.9 out of 1 (Figure 1E). In addition, the live-cell and dead-cell staining results revealed that the HepaRG spheroids seeded at a density of 5000 cells per well had a stronger dead signal inside the spheroid than those seeded at a density of 1000 cells per well (Figure 2B). This result is likely due to the increased risk of necrotic cores caused by hypoxia at spheroid diameters larger than 250 µm [29]. To facilitate the evaluation of the staining results, the red-fluorescence coverage area (dead cells) and the green-fluorescence coverage area (live cells) of individual spheroids were evaluated in a simple and accurate manner [30]. The percentage area of dead cells of the 1000-cell spheroid culture on day 7 was 1.8-fold higher than that on day 4 (Figure 2C,D), suggesting that the number of dead cells in the smaller spheroid slightly increased over time. While this outcome has not been previously reported in HepaRG spheroids, a study on the real-time viability detection of Cellosaurus tumor spheroids revealed that propidium iodide and caspase 3/7 fluorescence showed a time-dependent increase from day 4 to day 7 [31], which implies that the compaction-induced necrotic core was caused by hypoxia. The above results suggest that spheroids formed by 5000 cells are too large for in vitro 3D spheroid culture. Therefore, we also attempted to grow smaller HepaRG using 100-cell spheroids to check whether the percentage of dead cells could be reverted (Supplementary Figure S3). The results showed that a much improved round shape in 100-cell spheroids and the dead cells could be greatly attenuated compared to 1000- and 5000-cell spheroids, highlighting the crucial role of different spheroid sizes in maintaining proper waste, nutrients, and oxygen exchange. However, we found that growing 100 cells in each well of the 96-well ULA plate became less efficient and low-throughput in translating to the subsequent genomic analysis that required more plates and cost involved. In addition, the estimated radii of 5000-cell spheroids would be less precise due to its initial compaction process yielding a much irregular shape before day 4 compared to a more compacted and spherical structure formed in 1000-cell spheroids in day 3. Therefore, subsequent experiments were conducted with a more suitably sized spheroid composed of 1000 cells.

In the process of spheroid formation, cells undergo translocation, shape changes, and self-assembly; thus, cytoskeletal elements may play an important role in the spheroid formation. The lack of actin filaments in rat hepatocytes can lead to lower albumin secretion and reduce cytochrome P450 2B1/2 activities, confirming the indispensability of actin filaments for the 3D spheroid formation and hepatocyte functions [32]. To verify the actin filament content of HepaRG spheroids, immunofluorescence staining of actin filaments was performed. As shown in Figure 3B, the actin filaments of HepaRG spheroids had a dense network structure. This cell boundary marked the tight cell–cell connection of the spheroid structure, which showed that the 1000-cell spheroids formed a good 3D structure in the ULA 96-well plate. Our experimental results also confirmed that the in vitro HepaRG spheroids exhibited better hepatic function than traditional 2D-cultured cells, as revealed by the examination of hepatocyte-specific functional proteins through an immunofluorescence analysis of albumin (Figure 3A,B). In addition, the quantitative results indicated that the hepatic function of spheroids increased throughout the experimental period (Figure 3C). In some previous studies, 3D HepaRG spheroids had higher expression of hepatocyte-specific proteins than 2D HepaRG cells after 21 days of culture. These proteins include albumin, CYP1A2, and CYP3A4 [11]. The activities of the drug-metabolizing enzymes CYP1A2, CYP2B6, and CYP3A4 can reach physiologically relevant levels [14]. The long-term maintenance of hepatocyte functionality makes these models useful for liver toxicity screening, and the increase in albumin expression over time found in our study compared with the results for 21-day-old 3D HepaRG spheroids in other studies [11,14] shows that our in vitro 3D spheroid model has potential for application in drug toxicity screening. However, the development of phenotypic function depends on the culture duration, which inevitably affects the time needed for the experiment. Traditional 2D culture requires at least 4 weeks for HepaRG cells to differentiate and maximize liver-specific enzyme activities [13,33]. Other 3D culture methods also take time; for example, it took 14 days to encapsulate HepaRG cells in 1.5% alginate to obtain spheroids that met clinical needs [34].

With the aim of promoting HepaRG spheroid differentiation and shortening the time required for the formation of functional spheroids, we studied HepaRG spheroids embedded in rtCollagen or rhCollagen I. Morphology assessment showed that the spheroids embedded in either type of collagen type I continued to grow and be supported by a 3D ECM network by protruding from the periphery of the spheroids and growing outward to form a dendritic structure (Figure 4A). In addition, the embedded spheroids had appropriate viability, although the interior spheroids still presented a dead signal due to hypoxia and a lack of nutrients [29] (Figure 4B,D), and they had a good 3D structure according to the dense network structure of actin filaments (Figure 4C). The results from our functional validation experiment confirmed that HepaRG spheroids embedded in rtCollagen or rhCollagen I exhibited better hepatic function than spheroids grown in suspension, as demonstrated by the examination of hepatocyte-specific functional proteins through an immunofluorescence analysis of albumin and qPCR analysis of albumin and CYP3A4 (Figure 4E–G). These results indicate the important role of collagen I in promoting HepaRG differentiation and reducing the time needed for spheroid formation. The average fluorescence intensity of the spheroids embedded into rhCollagen was slightly higher than that of the spheroids embedded into rtCollagen, but there was no significant difference between the two values (Figure 4E). While whether rhCollagen I can better promote HepaRG differentiation than rtCollagen I remains to be confirmed, what is certain is that we can effectively shorten the time required to form functional spheroids by the addition of collagen I, and the choice of human recombinant collagen greatly paves a reliable source for reproducible and predicable ECM for human hepatocyte culture. Therefore, we envision this play a pivotal role in providing insights for the development of an in vitro liver model and for the assessment of drug toxicology. 

Despite the enabling and facile nature of the proposed method, there are some limitations that should be further addressed. First, although live/dead/immunofluorescence staining can be used as a facile method to rapidly observe the viability, spheroid structure, and albumin expression, enabling rapid quantification, it was found that signals from out-of-focus planes were also collected due to the nature of the widefield microscope. Therefore, applying a confocal microscope [35,36,37] that captures the light emitted in one focal plane is essential to perform an improved quantitative analysis (Supplementary Figure S4). Second, even though the levels of albumin and CYP3A4 expression increased in the proposed culture scaffold-free/scaffold-based conditions, this observation requires further investigation for improving the current model. For example, searching for a more comprehensive range of seeding densities may enable less initial nutrient consumption and enhanced viability of the spheroids grown in collagen I, identifying albumin secretion in the supernatant of the spheroids through ELISA. Third, the spheroids might be lost during the embedding process as the collagen I solution used in this study can be easily prepared by simply mixing various ingredients in proportions and is widely used. However, it is necessary to collect the spheroids in advance and then evenly mix the collected spheroids with collagen, which could lead to some loss of spheroids, and thus a reduction in their number during the process, highlighting a one-step method for the preparation of the collagen solution and the transfer of spheroids into the collagen solution, may be potential future research directions.

## 5. Conclusions

HepaRG spheroids formed in ULA 96-well plates showed a stable volume, a homogeneous spherical structure, and high cell viability throughout the experiment. The 3D spheroid culture expressed higher hepatocyte-specific functional proteins than traditional 2D culture, and the function increased in a time-dependent manner. Most importantly, the embedding of HepaRG spheroids with collagen I could accelerate HepaRG differentiation compared with that observed with spheroids grown in suspension. The culture conditions established in this study are believed to serve as a simple, economical, time-saving, and reproducible method for the generation of an in vitro hepatic model.

## Data Availability

Not applicable.

## Supplementary Materials

The following supporting information can be downloaded at: https://www.mdpi.com/article/10.3390/polym14091923/s1. Figure S1. The workflow for investigating the function of collagen I in promotion of liver differentiation in HepaRG spheroids; Figure S2. Schematic figures of applying ImageJ to calculate the live and dead cells’ area; Figure S3. Morphological and viability assessment 100-cell spheroids. (A) Aggregation processes of HepaRG spheroids with 100-cell concentration. Scale bar, 100 µm. (B) Live-cell and dead-cell staining of HepaRG spheroids. Scale bar, 50 µm; Figure S4. Immunofluorescence staining acquired by scanning confocal microscopy (FV3000, Olympus, Tokyo, Japan) of HepaRG 1000-cell spheroids at 7 days. Scale bar, 50 µm.
