# Supplementary material for: A Preliminary Investigation of Embedding In Vitro HepaRG Spheroids into Recombinant Human Collagen Type I for the Promotion of Liver Differentiation
Source: Polymers (Basel). 2022 May 9;14(9):1923. doi: 10.3390/polym14091923 (PMC9103061; doi:10.3390/polym14091923)
Supplement: Supplementary file 1 [file polymers-14-01923-s001.zip › polymers-1709817-supplementary.pdf]

Supplementary Material

1 Supplementary Figures

Experimental workflow for the investigate the function of collagen I in promotion of liver differentiation in HepaRG spheroids. At day 0, HepaRG cells were seeded in a 96-well plate and precultured for 4 days to form spheroids of 1000 cells. At day 4 of culture, the well-formed spheroids were harvested and mix with two types of collagen I (rat tail and humanized). The mixture of spheroids and collagen I was evenly distributed in each homemade PDMS chamber and maintained for 3 days (day 7 of culture). After 7 days of culture, spheroids were be assessed through live/dead staining and immunofluorescence microscopy.

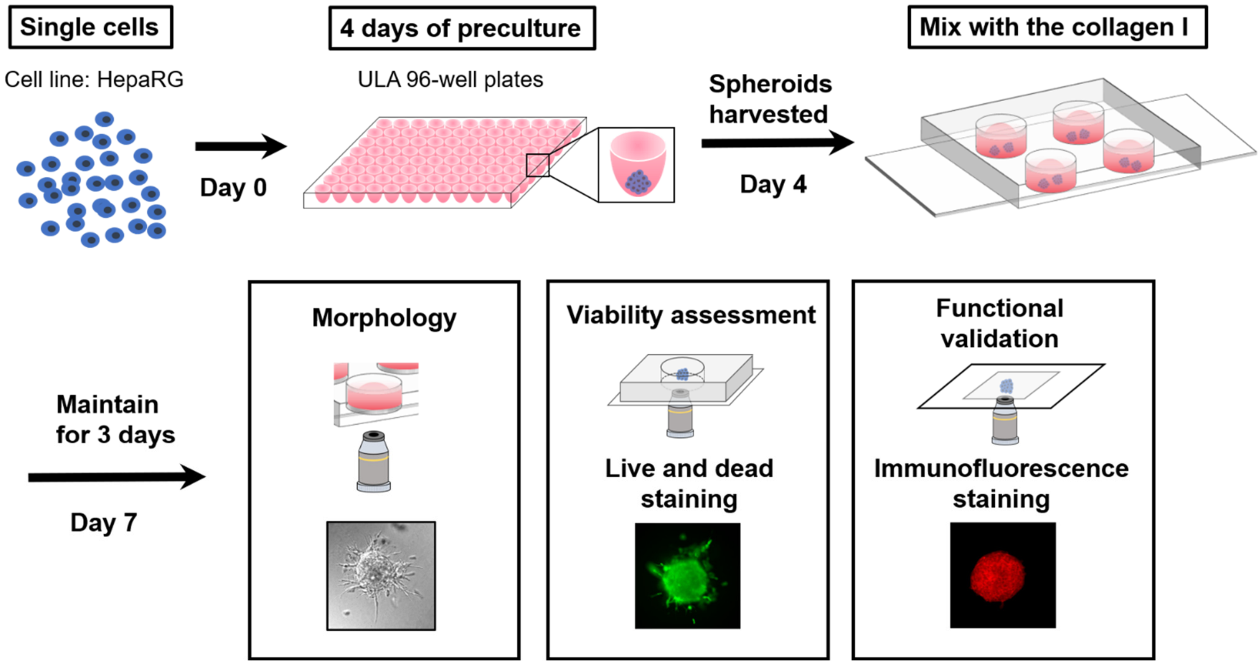

Figure S1. The workflow for investigating the function of collagen I in promotion of liver differentiation in HepaRG spheroids.

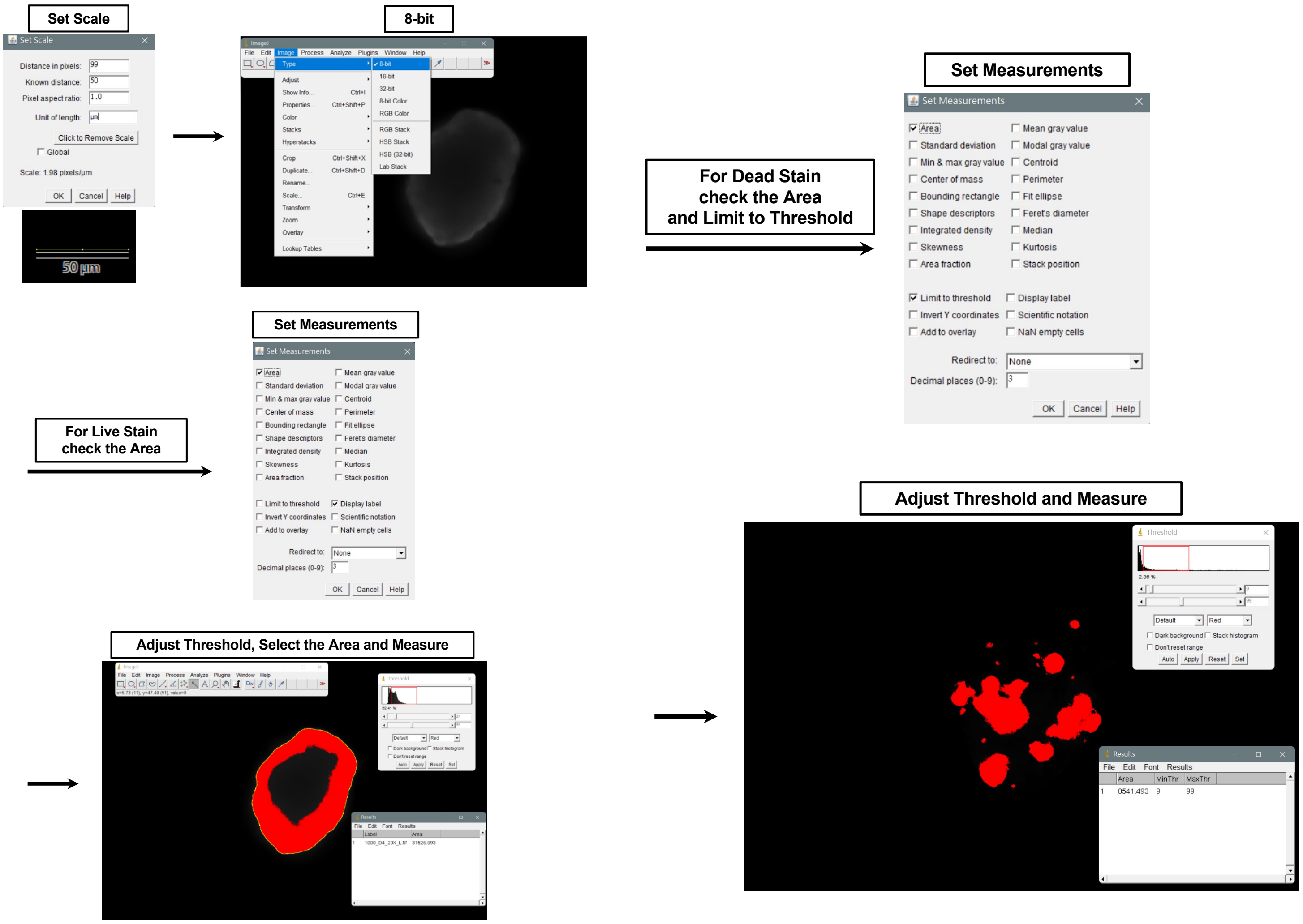

Figure S2. Schematic figures of applying ImageJ to calculate the live and dead cells' area.

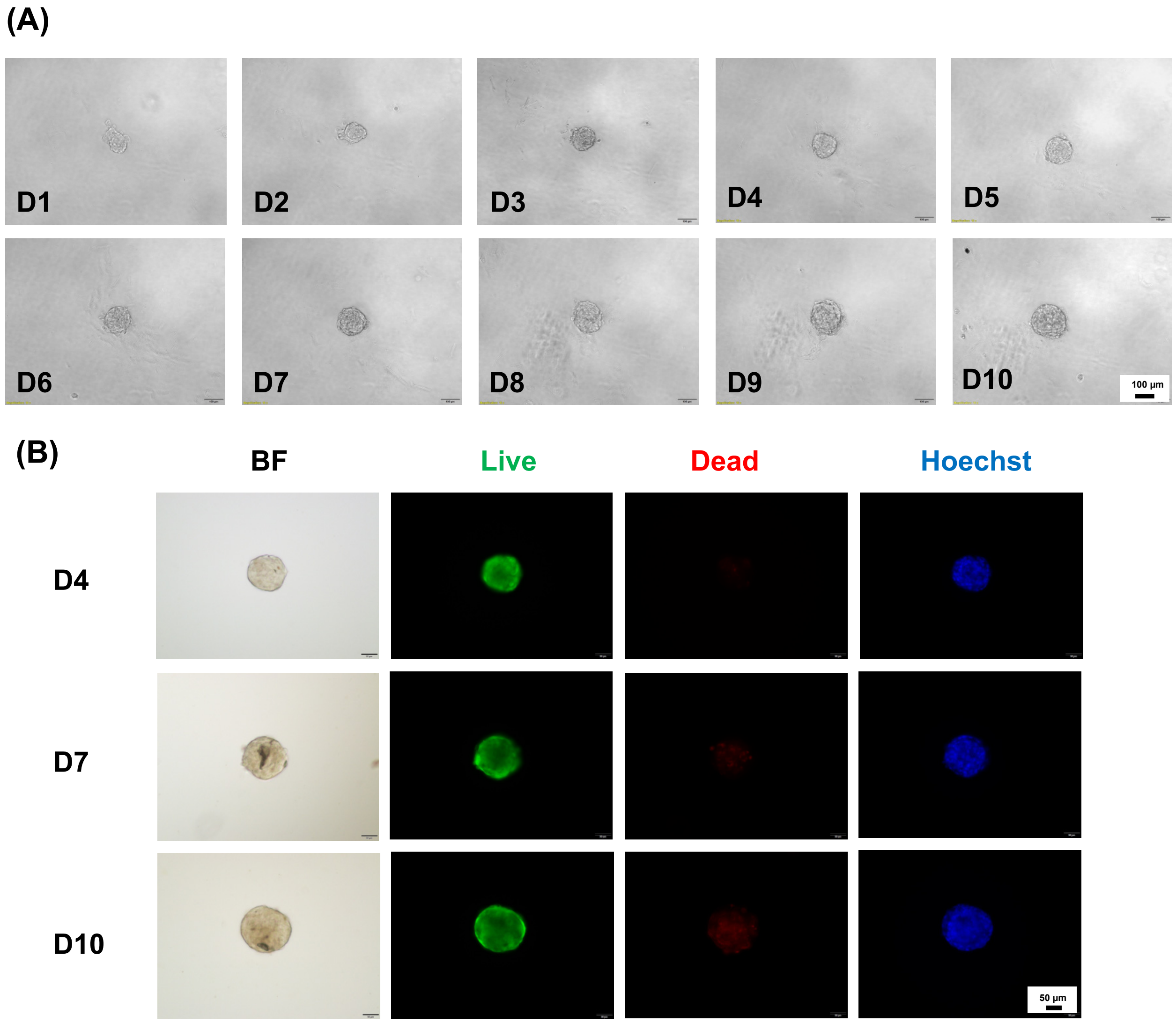

Figure S3. Morphological and viability assessment 100-cell spheroids. **(A)** Aggregation processes of HepaRG spheroids with 100-cell concentration. Scale bar, 100 µm. **(B)** Live-cell and dead-cell staining of HepaRG spheroids. Scale bar, 50 µm.

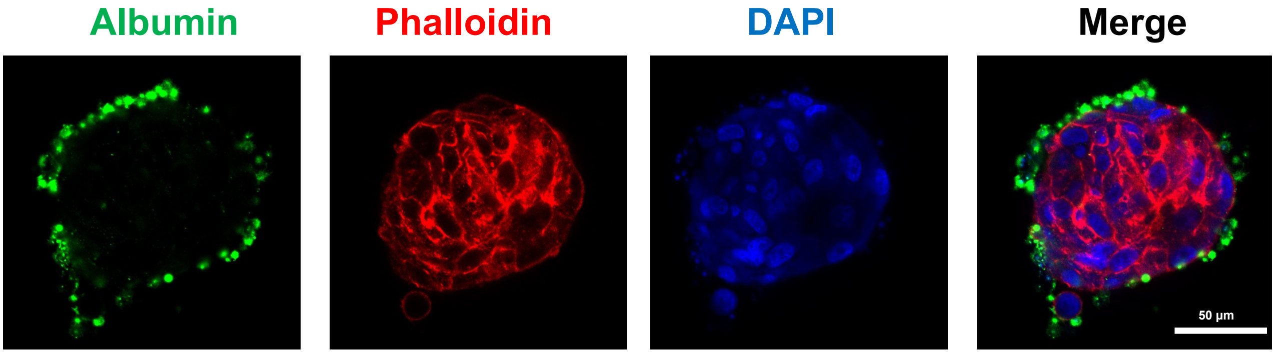

Figure S4. Immunofluorescence staining acquired by scanning confocal microscopy (FV3000, Olympus, Tokyo, Japan) of HepaRG 1000-cell spheroids at 7 days. Scale bar, 50 µm.
